# Subtype-specific NK cell-TAM interactions drive a novel prognostic signature in HNSCC

**DOI:** 10.3389/fimmu.2025.1676878

**Published:** 2025-10-17

**Authors:** Zhenyan Zhao, Xuejiao Han, Yating Hu, Yun Li, Yaodong He, Yan Wang, Yanbing Yao, Huan Li, Jianhua Wei

**Affiliations:** State Key Laboratory of Oral and Maxillofacial Reconstruction and Regeneration, National Clinical Research Center for Oral Diseases, Shaanxi Clinical Research Center for Oral Diseases, Department of Oral and Maxillofacial Surgery, School of Stomatology, The Fourth Military Medical University, Xi’an, China

**Keywords:** head and neck squamous cell carcinoma, tumor microenvironment, NK cells, tumor-associated macrophages, cell interaction, prognostic model

## Abstract

**Background:**

The immune microenvironment of head and neck squamous cell carcinoma (HNSCC) is highly complex, and the mechanisms underlying interactions between natural killer (NK) cells and tumor-associated macrophages (TAMs) remain unclear. This study investigates the cellular heterogeneity, interaction patterns, and prognostic significance of NK-TAM crosstalk through multi-omics analyses.

**Methods:**

A total of 58 HNSCC tissue samples were analyzed. NK and TAM subsets were identified using immunohistochemistry (CD16, CD64, CD163), single-cell RNA sequencing (GSE139324), and public databases (TCGA-HNSC, GSE65858). CellChat was used to infer ligand-receptor interactions, while spatial proximity was assessed via the CSOmap algorithm and validated by immunofluorescence. A prognostic model was constructed using LASSO Cox regression and validated in an immunotherapy cohort (PRJEB23709, phs000452.v2.p1).

**Results:**

High CD16/CD64 expression correlated with favorable prognosis, while CD163 indicated poor outcomes (*P* < 0.05). NK cells were divided into IL32+NK (antiviral, T cell–activating), NFKBIA+NK (ribosome-related), and STMN1+NK (DNA repair–related) subsets. TAMs included APOE+TAM (M2-like), IL1B+/CXCL10+TAM (M1-like), and HSP+TAM (stress-responsive). IL32+NK interacted most strongly with APOE+TAM and CXCL10+TAM via SPP1, MIF, and ITGB2 pathways. Spatial mapping and immunofluorescence confirmed proximity and a positive correlation between IL32 and CXCL10 (R = 0.641, *P* < 0.001), and a negative correlation with APOE (R=–0.686, *P* < 0.001). A 23-gene NK-TAM interaction–related signature (CINT) effectively stratified patient risk in both training and validation cohorts (*P* < 0.05) and predicted survival benefit in immunotherapy-treated patients.

**Conclusion:**

This study uncovers subtype-specific NK-TAM interactions in HNSCC and introduces CINT as a robust prognostic and immunotherapy response model, offering a new strategy for immune microenvironment–targeted therapy.

## Introduction

Head and neck cancer (HNC) is the seventh most prevalent cancer worldwide, with approximately 890,000 new cases and 450,000 deaths annually (1). Among HNCs, head and neck squamous cell carcinomas (HNSCCs) are the predominant histological subtype (2), representing over 90% of all cases. The incidence and prevalence of HNSCC vary considerably across different countries and regions. These disparities are primarily linked to factors such as tobacco exposure, excessive alcohol consumption, and human papillomavirus (HPV) infection (3, 4). Standard treatment options for head and HNSCC currently encompass surgery, radiotherapy, chemotherapy, immunotherapy, or a combination of these modalities. Immunotherapy, in particular, has gained attention as a promising therapeutic avenue for HNSCC (5). The immune microenvironment significantly influences the pathophysiology of the disease (6). To improve patient prognosis and the effectiveness of immunotherapy for HNSCC, it is crucial to conduct in-depth exploration of the tumor immune microenvironment.

NK cells, a key component of the innate immune system, play a vital role in eliminating virally infected, stressed, and malignant cells. Human NK cells are classified into two subsets based on their surface expression of CD56: CD56^bright^ and CD56^dim^, each exhibiting distinct phenotypic traits (7). CD16 is a key functional marker of NK cells, particularly highly expressed in the CD56^dim^ subset, which mediates antibody-dependent cellular cytotoxicity (ADCC). It serves as a central target for enhancing the anti-tumor activity of NK cells. Activation of NK cells occurs through various molecular signals relayed by stimulatory or inhibitory receptors found on a range of immune cells, including macrophages, dendritic cells, eosinophils, and T cells. This activation not only enables NK cells to execute their cytotoxic functions but also facilitates communication and co-stimulation, allowing them to modulate both innate and adaptive immune responses effectively (8, 9).

TAMs are functionally categorized into classically activated M1 and alternatively activated M2 types (10). Human M1 macrophages, marked by CD86 and CD64, act as the first line of defense against microbial infections, exhibiting strong antigen-presenting capacity and eliciting robust Th1 responses. M2 macrophages, characterized by the expression of surface markers such as CD206 and CD163 (11), play a crucial role in modulating immune responses. As a result, M2-type TAMs are frequently associated with pro-tumor activities, whereas M1-type TAMs are linked to anti-tumor effects (10, 12). This functional dichotomy highlights the importance of understanding the balance between these macrophage subsets within the tumor microenvironment, as it influences both tumor progression and therapeutic outcomes.

Emerging evidence highlights the complex crosstalk between TAMs and NK cells, a critical determinant of anti-tumor immune responses. IL-10 secreted by TAMs suppresses the local production of IL-12, a cytokine essential for inducing a Th1 response and enhancing NK cell cytotoxicity (13). Young et al. (14) also demonstrated that macrophages can inhibit NK cell function, showing that prostaglandin E2 (PGE2) released by alveolar macrophages suppressed NK cell activity in a murine lung carcinoma model. Both M2-polarized macrophages and TAMs inhibited NK cell CD27 expression and cytotoxicity in a contact-dependent manner, with TGF-β being essential for the suppressive effect of M2 macrophages. Additionally, TAMs promoted a CD27^low^ CD11b^high^ exhausted NK cell phenotype (15). However, the functional interactions between specific subsets of NK and TAMs and their prognostic significance in the microenvironment of HNSCC remain unclear. This study aims to focus on the key cell interaction groups in the microenvironment of head and neck squamous cell carcinoma, especially the NK and TAM cell subpopulations with special status, and explore the special status and prognostic efficacy of cell subpopulations from the perspective of cell interaction.

## Materials and methods

### Patients and specimens

A total of 58 primary HNSCC specimens were collected from the tissue bank of our affiliated hospital from 2014 to 2019. All patients underwent radical surgical resection according to the NCCN guidelines and did not receive chemotherapy or radiotherapy before surgery. Follow-up was performed by telephone interview or medical record review. Clinical pathological parameters, including tumor stage, degree of differentiation, smoking and drinking history, were retrieved from medical records and follow-up data. The present study was approved by the Medical Research Ethics Committee of The Fourth Military Medical University.

### Immunohistochemical staining

Paraffin-embedded tumor tissue samples were collected for immunohistochemical (IHC) analysis. Primary antibodies included rabbit polyclonal anti-CD16 (Servicebio, GB113963), rabbit polyclonal anti-CD163 (Servicebio, GB115709), and rabbit monoclonal anti-CD64 (Abcam, ab302901). Peroxidase-conjugated goat anti-rabbit IgG antibodies were used as secondary antibodies. All stained sections were independently and blindly evaluated by two experienced pathologists. Tumor samples exhibiting positive staining rates above the median were classified as having high expression, while those with staining rates at or below the median were classified as having low expression.

### Data collection

FPKM expression profiles for TCGA-HNSC were downloaded using the R package TCGAbiolinks, followed by log transformation. Survival data and clinical information were also collected, retaining 494 tumor samples with both expression and survival information for signature construction.

The GEO database (https://www.ncbi.nlm.nih.gov/geo/) was used to download bulk expression profile data from GSE65858 along with corresponding clinical information for signature validation. The data processing standards for GEO bulk datasets involved converting probes to symbols based on the correspondence of each platform. Probes corresponding to multiple genes were removed, while for multiple probes corresponding to the same symbol, the median value was taken.

Additionally, the GSE139324 single-cell dataset for HNSCC was downloaded from the GEO database. This dataset includes expression profiles from 32 peripheral blood samples (26 tumor + 6 normal) and 31 tissue samples (26 tumor + 5 normal). Among these, 31 tissue samples were selected for this project analysis, focusing exclusively on immune cells (specifically NK cells and TAMs) as required by the study design.

Clinical and transcriptomic data from two cohorts of tumor patients undergoing PD-1/PD-L1 blockade therapy (PRJEB23709 and phs000452.v2.p1, Van Allen et al.) were downloaded to evaluate the predictive efficacy of the signature in immunotherapy cohorts. Details of the data types, actual sample sizes used in the analysis, and their respective purposes are summarized in the **Table 1**.

**Table 1:** Datasets used in this study.

Dataset ID	Data Type	Number of Tumor Samples Used	Purpose of Analysis
TCGA-HNSC	bulk	494	Signature construction; training set
GSE65858	bulk	270	Signature validation; validation set
PRJEB23709	bulk	90	Evaluation of signature predictive performance in immunotherapy cohorts
phs000452.v2.p1	bulk	41	Evaluation of signature predictive performance in immunotherapy cohorts
GSE139324	scRNA	26 tumor / 5 normal	Identification of NK cell subpopulations, TAM subpopulations, and their marker genes

### Single-cell transcriptomic data quality control

Quality control of the 31 single-cell samples was performed using the R package Seurat (v4.1.0). To exclude low-quality cells and low-expressed genes, the following thresholds were set: (1) each gene must be expressed in at least 3 cells; (2) the number of features per cell was restricted to between 500 and 2000, and the number of counts per cell was set between 1000 and 7500; (3) the proportions of mitochondrial genes and red blood cell genes in each cell were both limited to less than 10%.

Subsequently, the NormalizeData function was used for normalization, and the FindVariableFeatures function was employed to identify highly variable genes based on an average expression value (greater than 0.1 and less than 8) and dispersion (greater than 0.5). Batch correction between samples was conducted using the R package Harmony to avoid batch effects interfering with downstream analyses. The data were then scaled, and dimensionality reduction was performed using principal component analysis (PCA), selecting the top 50 principal components for downstream analysis. Visualization was accomplished using the RunTSNE function, and cell clustering was conducted using the FindClusters function. Cell types were annotated based on the expression of known markers.

### Identification of NK cell subpopulations

NK cells were extracted from tumor samples, and following standardization, normalization, identification of highly variable genes, batch effect correction, and PCA (with parameters consistent with the data quality control section), the top 50 principal components were selected with a resolution set to 0.3. Clustering and subgroup identification were performed to recognize NK cell subpopulations. Characteristic genes for each subpopulation were identified using the FindAllMarkers function (with avg_log2fc > 0.25 and p_val_adj < 0.05).

### Identification of TAM cell subpopulations

Myeloid cells were extracted from tumor samples, and similar procedures of standardization, normalization, identification of highly variable genes, batch effect correction, and PCA were applied (with parameters consistent with the data quality control section). The top 50 principal components were selected, and a resolution of 0.3 was set for clustering and subgroup identification. Based on cell type markers, four TAM subpopulations, two DC subpopulations, and two monocyte subpopulations were identified. Characteristic genes for each subpopulation were identified using the FindAllMarkers function (with avg_log2fc > 0.25, p_val_adj < 0.05, and min.pct > 0.5).

### Cell communication analysis

To investigate the potential interactions between NK cell subpopulations and TAM cell subpopulations, cell communication analysis was performed on single-cell data using the R package CellChat. The specific steps are as follows: the CellChat object was constructed using the create CellChat function, and cell subpopulations were set as the default cell identifiers using the setIdent function. The CellChatDB.human database was configured as the ligand-receptor interaction database. Overexpressed genes were identified using the identifyOverExpressedGenes function, and overexpressed ligand-receptor interactions were identified using the identify OverExpressedInteractions function. The gene expression data were projected onto the protein-protein interaction (PPI) network using the projectData function. Communication probabilities were calculated and the CellChat network was inferred using the computeCommunProb function. Filtering was performed with the filterCommunication function, setting min.cells = 10 as the threshold. Cell-cell communication was inferred at the signaling pathway level using the computeCommunProbPathway function, and the communication network was aggregated using aggregateNet. Subsequently, the interaction counts among different cell groups were visualized using the netVisual_circle function. Additionally, the network centrality scores were calculated using netAnalysis_computeCentrality, and the visualization of the centrality scores was conducted with the netAnalysis_signalingRole_network function.

### Immunofluorescence analysis

Immunofluorescence (IF) staining was performed on 58 paraffin-embedded HNSCC tissue sections (4 μm thick) using the tyramide signal amplification (TSA) system. Sections were deparaffinized in xylene, rehydrated in graded ethanol, and antigen-retrieved via microwave heating in EDTA buffer (pH 8.0). Endogenous peroxidase was quenched with 3% H_2_O_2_ for 10 min, followed by blocking with 5% BSA or 10% goat serum for 30 min. The slides were then incubated overnight at 4°C with primary antibodies, including anti-APOE monoclonal antibody (66830-1-Ig,Proteintech,1:400), anti-IL-32 polyclonal antibody (11079-1-AP, Proteintech,1:400), and anti-CXCL10 polyclonal antibody (10937-1-AP, Proteintech,1:500). The next day, sections were washed in PBS and incubated with horseradish peroxidase (HRP)-conjugated secondary antibodies, followed by TSA fluorophore development according to the manufacturer’s instructions. Nuclear staining was performed using 4′,6-diamidino-2-phenylindole (DAPI). For multiplex staining, antigen retrieval and antibody incubation steps were repeated for each target protein. After autofluorescence quenching, slides were mounted with anti-fade medium. Images were captured using a Nikon Eclipse C1 fluorescence microscope and analyzed with Fiji ImageJ software.

### Spatial organization and communication of cells

The spatial organization of cells is closely related to various cellular functions and behaviors, including cell-to-cell interactions. However, scRNA-seq data typically lack such spatial information, as cells must be separated prior to sequencing. The CSOmap algorithm developed by Zhang Zemin’s team enables the spatial reconstruction of gene expression using only scRNA-seq data (16). Specifically, CSOmap can not only predict cell interactions but also infer cellular spatial organization from scRNA-seq data, construct spatial expression patterns of ligands and receptors, and infer intercellular communication information, revealing signaling mechanisms of cell behaviors such as tumor progression, development, and differentiation. Therefore, the CSOmap algorithm was utilized to infer the three-dimensional proximity and communication information between NK cell subpopulations and TAM cell subpopulations.

### Construction and validation of prognostic biomarkers based on cellular interaction mechanisms

Based on the characteristic genes of NK cell subpopulations, the ssGSEA algorithm from the R package GSVA was used to calculate the enrichment scores of each cell subpopulation for TCGA-HNSC samples as subpopulation features. Univariate Cox regression analysis was performed to determine the hazard ratios (HR) and prognostic significance of the characteristics of each NK cell subpopulation.

We integrated cell communication and spatial data to identify TAM populations that significantly interact with prognosis-related NK cell subpopulations (*p* < 0.05) and are spatially accessible. The characteristic genes of these interacting TAM-NK cell groups were then selected as candidate genes for further analysis.

Univariate Cox regression analysis was then used to determine the HR and prognostic significance of the candidate genes, filtering for genes with *p* < 0.05 to identify prognostic-related genes. LASSO regression analysis (using the R package glmnet) was employed to further select key prognostic factors. A risk score signature for predicting patient survival was constructed by weighting the expression of each key prognostic factor with its corresponding LASSO regression coefficient (where “exp” represents gene expression levels and “coef” represents LASSO regression coefficients):


$$\text{Score}=\sum_{i=1}^{n}exp_{i}\times coef_{i}$$

Samples were divided into high and low groups based on their scores. Kaplan-Meier survival curves were generated for prognostic analysis, and the log-rank test was utilized to determine the significance of differences between the two groups, further analyzing the correlation of these two categories with OS. The predictive capability of the scoring system was evaluated using receiver operating characteristic (ROC) curves, with the area under the curve (AUC) visualized using the R package timeROC. Additionally, both univariate and multivariate Cox analyses were conducted to explore the independent prognostic value of the score.

### GSVA (gene set variation analysis) and functional enrichment

GSVA is a non-parametric, unsupervised method primarily used to estimate variations in pathway and biological process activity within samples. Gene sets from the KEGG and GOBP sub-libraries of the MSigDB database were downloaded for GSVA analysis. The R packages GSVA and GSEABase were utilized to compare functional differences among different cell subpopulations.

### Statistical analysis

All *in vitro* experiments were independently repeated three times. Intergroup comparisons and associations between protein expression and clinicopathological parameters were analyzed using the χ² test. Correlations between protein expression levels were assessed using Pearson correlation analysis. Survival analyses were conducted using the Kaplan–Meier method, with differences compared using the log-rank test. Statistical analyses were performed using SPSS software (version 24.0; IBM Corp., Armonk, NY, USA), and a two-tailed P value < 0.05 was considered statistically significant.

Bioinformatics analyses were carried out using R software (version 4.1.2). For comparisons of expression levels, invasion rates, and other characteristics, the Wilcoxon rank-sum test was used for two-group comparisons, while the Kruskal–Wallis test was applied for comparisons among multiple groups. In the figures, “ns” indicates *P* > 0.05, * indicates *P* < 0.05, ** indicates *P* < 0.01, *** indicates *P* < 0.001, and **** indicates *P* < 0.0001.

## Results

### Correlation between CD16, CD64 and CD163 expressions and patient prognosis

As summarized in **Table 2**, CD16, CD64, and CD163 exhibited no significant association with the evaluated clinicopathological parameters, encompassing gender, age, smoking history, alcohol history. CD16 and CD64 exhibit high expression in early-stage disease and well-differentiated tumors, in contrast to CD163, which is upregulated in advanced stages and poorly differentiated tumors.

**Table 2:** Clinicopathological correlates of CD16, CD64, and CD163 expression.

Variables	N	CD16		χ²	P-value	CD64		χ²	P-value	CD163		χ²	P-value
		High(n=31)	Low(n=27)			High(n=30)	Low(n=28)			High(n=26)	Low(n=32)		
Gender													
Male	25	13	12	0.037	0.847	14	11	0.007	0.933	12	13	0.179	0.672
Female	33	18	15			16	12			14	19		
Age													
≤50	30	16	14	0.000	0.849	15	15	0.074	0.786	12	18	0.586	0.444
>50	28	15	13			15	13			14	14		
Stage													
I + II	24	17	7	4.973	0.026^*^	17	8	5.107	0.024^*^	7	17	4.060	0.044^*^
III + IV	34	14	20			13	21			19	15		
Smoking history													
Nonsmoker	41	22	19	0.002	0.960	21	20	0.025	0.874	19	22	0.130	0.719
Smoker	17	9	8			9	8			7	10		
Alcohol history													
Nondrinker	45	25	20	0.358	0.549	22	23	0.014	0.905	21	24	0.275	0.600
Drinker	13	6	7			8	5			5	8		
Pathological differentiation													
Well	23	17	6	6.416	0.011^*^	18	5	10.75	0.001^*^	6	17	5.142	0.020^*^
Moderately/poorly	35	14	21			12	23			20	15		

**Figure 1:**
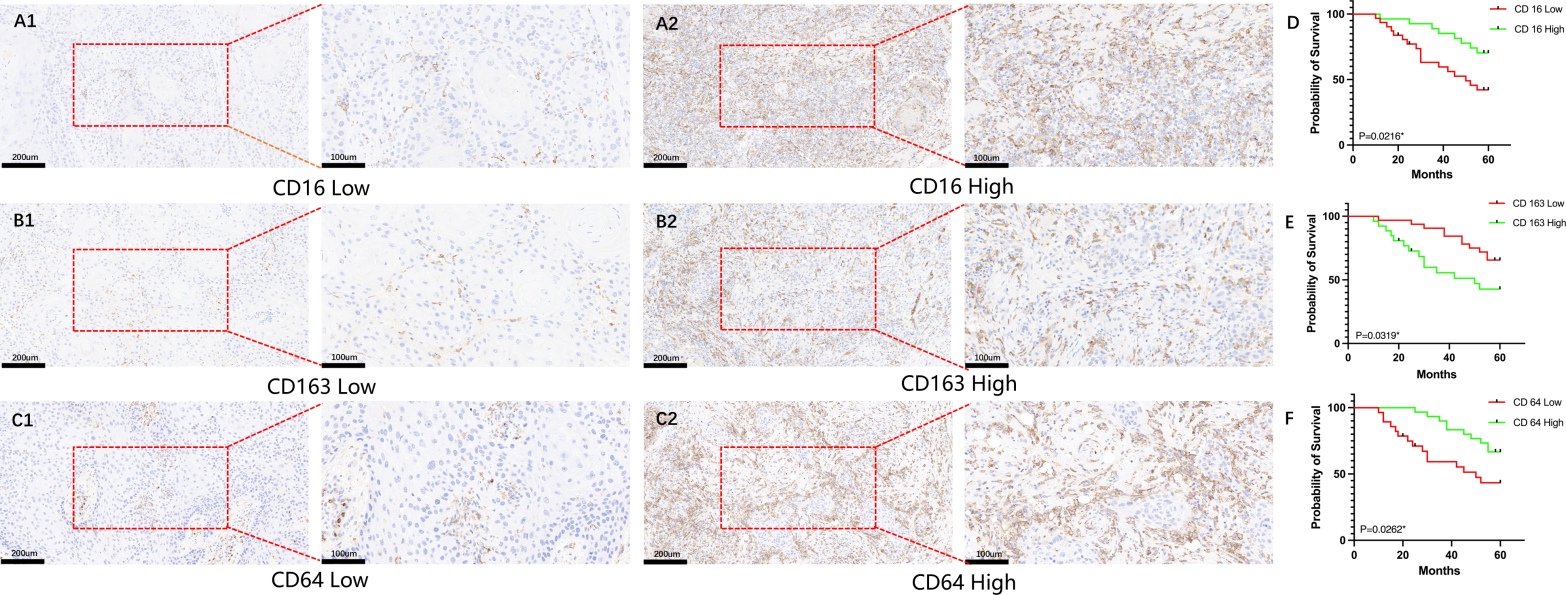
Analysis of the impact of CD16, CD64, and CD163 expression on survival in HNSCC patients. **(A1–C1)** Representative immunohistochemical images showing low-level expression of CD16, CD64, and CD163 in HNSCC patients; **(A2–C2)** Representative immunohistochemical images showing high-level expression of CD16, CD64, and CD163 in HNSCC patients; **(D, F)** High-level expression of CD16 and CD64 is significantly associated with improved overall survival in HNSCC patients (P < 0.05); **(E)** High-level expression of CD163 is significantly associated with poorer overall survival in HNSCC patients (P < 0.05).

All patients were followed until death or for a maximum of 60 months. By the end of the follow-up period, 4 patients (6.9%; 4/58) were lost to follow-up, 29 patients (50.0%; 29/58) were alive, and 25 patients (43.1%; 25/58) had died. The overall survival rate of patients with HNSCC was analyzed based on the expression levels of CD16, CD64, and CD163. As shown in **Figure 1**, high expression of CD16 and CD64 was significantly associated with better prognosis in HNSCC patients (*P* < 0.05; **Figures 1A1, A2, C1, C2**), whereas high expression of CD163 correlated with poorer prognosis (*P* < 0.05; **Figures 1B1, B2, E**).

### Microenvironment cell landscape

**Figure 2:**
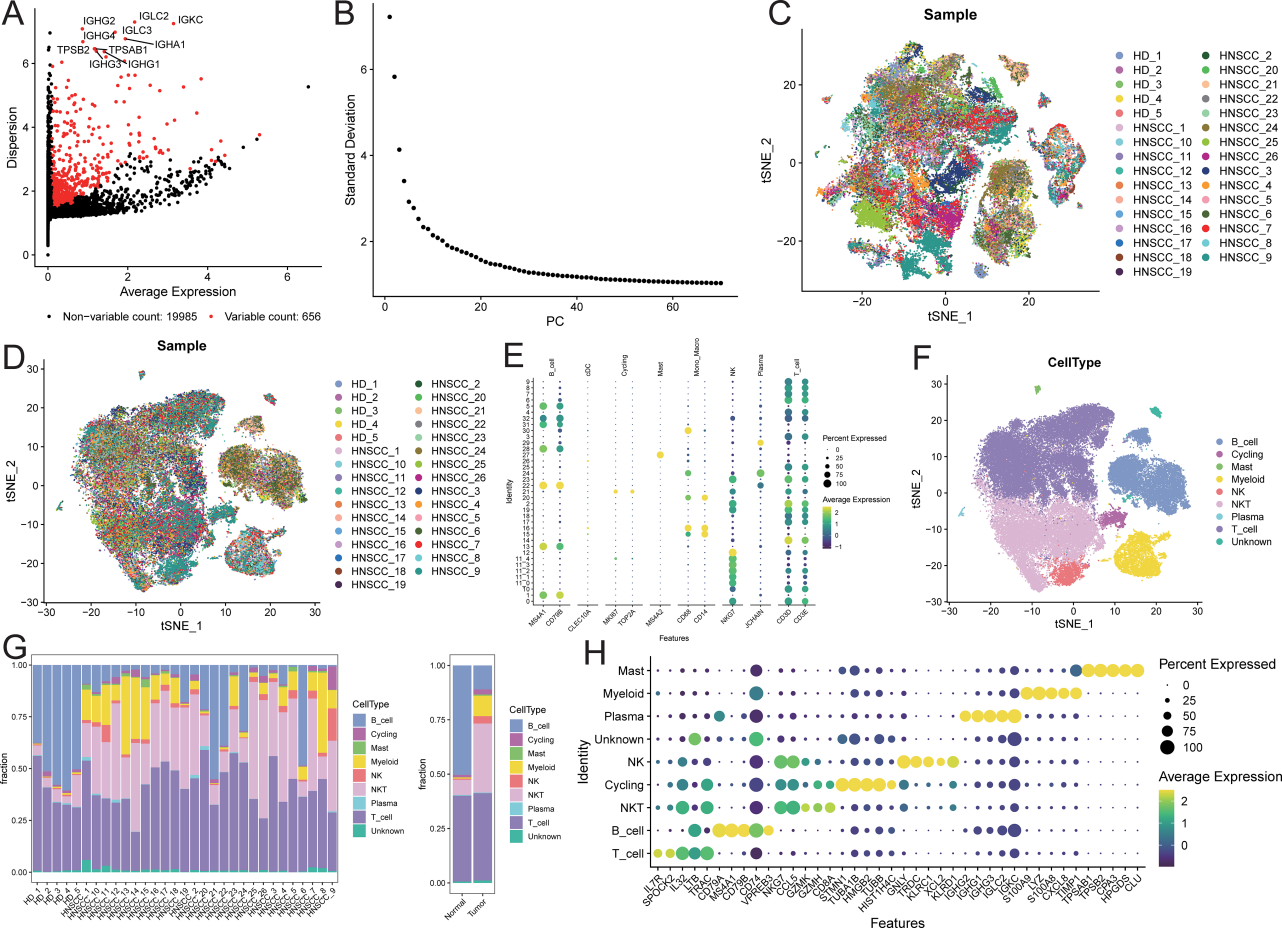
Microenvironment cell landscape. **(A)** scatter plot of highly variable genes; **(B)** ElbowPlot of principal component analysis; **(C)** TSNE distribution of sample cells before removing batch effect; **(D)** TSNE distribution of sample cells after removing batch effect; **(E)** bubble plot of marker gene expression; **(F)** TSNE distribution of cell types; **(G)** distribution ratio of sample cells; **(H)** bubble plot of top5 characteristic gene expression.

After quality control of the single-cell data, a total of 60,951 cells were retained, detecting 20,641 genes (quality control results are shown in **Supplementary Figure S1**). Among these, 656 genes were defined as highly variable genes (**Figure 2A**), including IGHG2, IGHG3, and IGHG1. The top 50 principal components (PCs) were selected for subsequent t-SNE visualization analysis (**Figure 2B**), and Harmony was used to remove batch effects between samples (**Figures 2C, D**). At a resolution of 2, 32 + 5 cell clusters were identified (**Supplementary Figure S2**). Cell types for each cluster were annotated based on known markers (**Figure 2E**; marker expression t-SNE distribution is shown in **Supplementary Figure S2**). The identified cell populations included 11,069 B cells, 1,287 cycling cells, 290 mast cells, 4,778 myeloid cells, 1,747 NK cells, 16,570 NKT cells, 206 plasma cells, 24,409 T cells, and 595 unknown cells (**Figure 2F**; t-SNE distribution maps of the two sample groups are shown in **Supplementary Figure S3**). The cell proportions in the two sample groups were statistically analyzed (**Supplementary Table S1**), revealing that the proportions of myeloid, NKT, NK, and mast cells were higher in tumor samples compared to normal samples, while the proportion of B cells was lower in tumor samples (**Figure 2G**; see **Supplementary Figure S4**). Subsequently, characteristic genes for different cell types were identified based on FindAllMarkers (**Supplementary Table S2**), sorted by avg_log2FC in descending order, and filtered to retain genes expressed in at least 50% of the target subpopulation. The top 5 characteristic genes for each cell group included LYZ, MS4A1, GNLY, among others (**Figure 2H**).

### Identification of NK cell subpopulations

**Figure 3:**
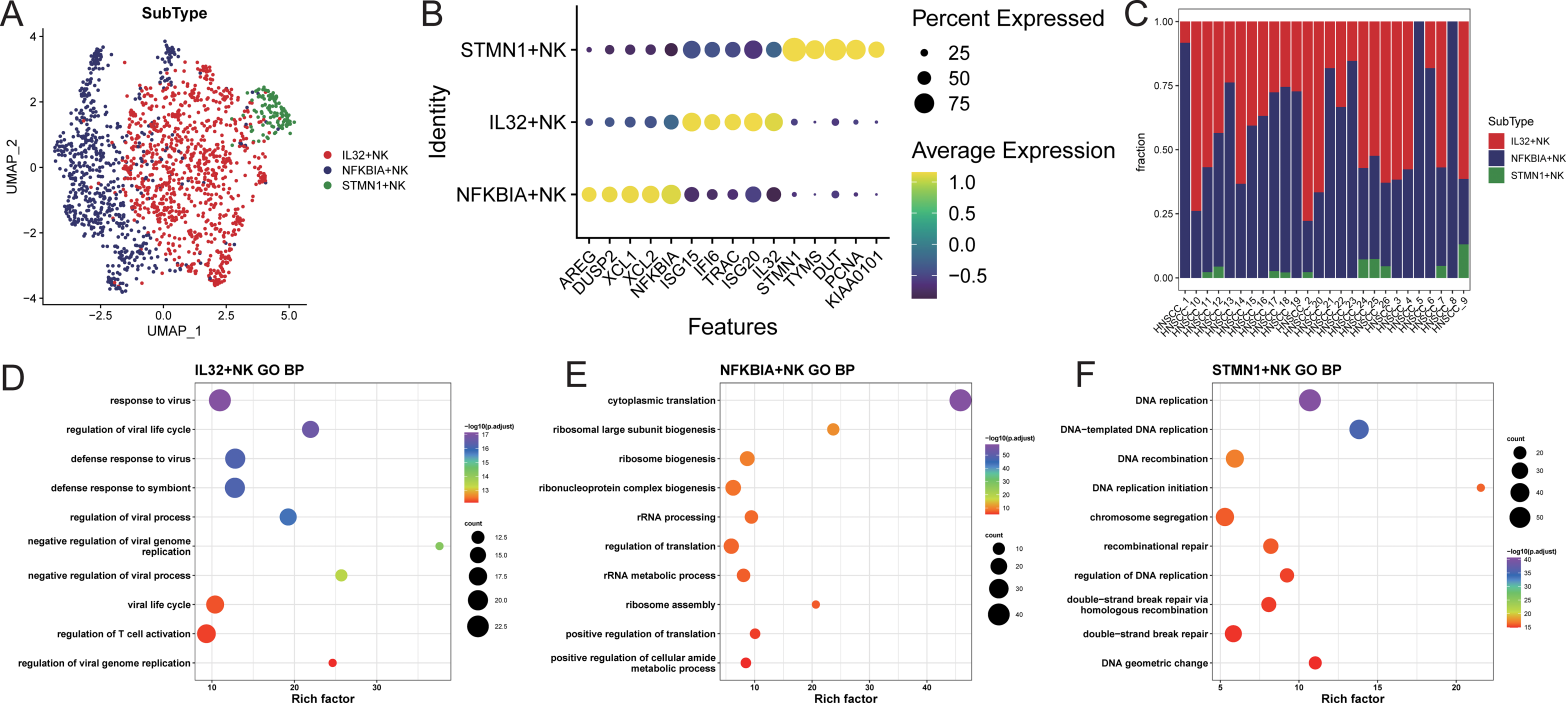
NK cell subset identification. **(A)** UMAP distribution of cells in each subpopulation; **(B)** bubble chart of top5 characteristic gene expression; **(C)** distribution ratio of cell subpopulations in each sample; **(D–F)** GOBP enrichment analysis bubble chart of characteristic genes in each subpopulation.

NK cells were extracted and re-normalized for clustering analysis, resulting in the identification of three cell clusters (**Supplementary Figure S5A**). Characteristic genes for each cluster were identified using FindAllMarkers, and subpopulations were named based on the highly expressed genes of each cluster (**Supplementary Figure S5B**; **Figure 3B**). The identified subpopulations included IL32+NK (n=904), NFKBIA+NK (n=675), and STMN1+NK (n=112; **Figure 3A**), with varying proportions of cell subpopulations across samples (**Figure 3C**; **Supplementary Table S3**). Characteristic genes for each NK cell subpopulation were identified using FindAllMarkers (**Supplementary Table S4**). The IL32+NK subpopulation exhibited high expression of genes such as ISG15, TRAC, and ISG20, with its characteristic genes enriched in biological processes related to responses to viruses and exogenous stimuli, as well as T cell activation regulation (**Figure 3D**; **Supplementary Table S5**). The NFKBIA+NK subpopulation expressed high levels of AREG, XCL1, and XCL2, with characteristic genes enriched in biological processes related to cytoplasmic translation, nucleolar assembly, and ribonucleoprotein complex biogenesis (**Figure 3E**; **Supplementary Table S5**). The NFKBIA+NK subset exhibited concomitant elevation of XCL1 and XCL2, chemokines critical for recruiting cDC1 to bridge innate and adaptive immunity (17), together with AREG, a factor involved in tissue repair and immune regulation (18). This expression profile suggests that these cells may function as an immunoregulatory NK population specialized in coordinating antitumor immunity and modulating the tumor microenvironment.The STMN1+NK subpopulation showed high expression of TYMS, DUT, and PCNA, with characteristic genes enriched in biological processes such as DNA replication, DNA recombination, and repair mechanisms (**Figure 3F**; **Supplementary Table S5**). The concomitant high expression of PCNA (a canonical marker of cell proliferation (19)), along with TYMS and DUT (key enzymes involved in deoxyribonucleotide synthesis and genomic fidelity (20, 21)), indicates that the STMN1+NK subset may represents a proliferative NK cell population poised for clonal expansion, potentially contributing to the maintenance and renewal of NK cells within the tumor microenvironment.

Using TCGA-HNSC samples, feature scores for each NK cell subpopulation were calculated, and samples were stratified into high and low feature groups based on these scores. The results indicated that the high feature group of IL32+NK was associated with better prognosis (**Supplementary Figure S6**).

### Identification of TAM cell subpopulations

**Figure 4:**
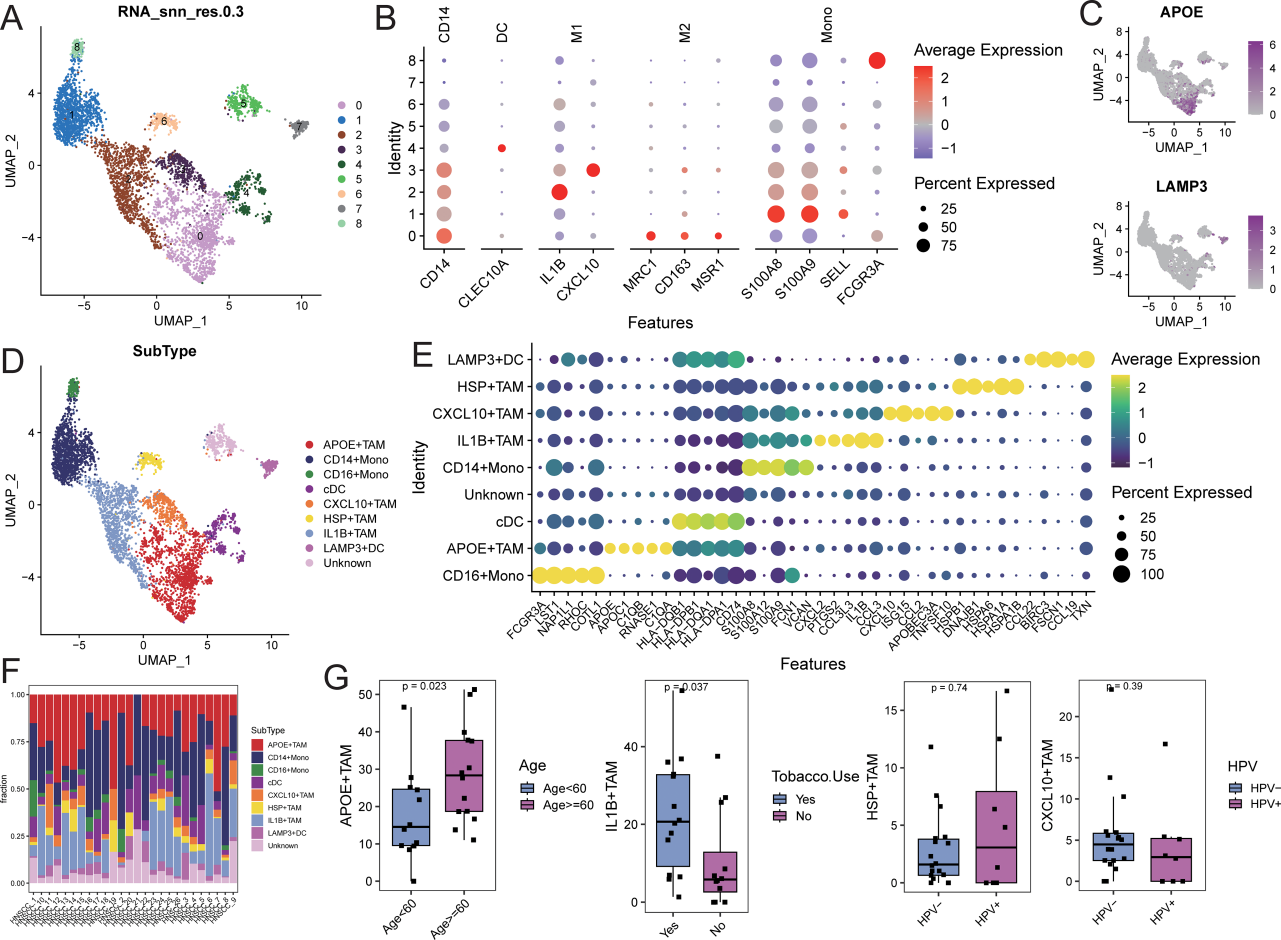
Identification of myeloid cell subsets. **(A)** UMAP distribution of different cell clusters; **(B)** Bubble diagram of marker gene expression; **(C)** UMAP distribution diagram of marker gene expression; **(D)** UMAP distribution of cells in each subpopulation; **(E)** Bubble diagram of top5 characteristic gene expression; **(F)** Proportion of cell subpopulation distribution in each sample; **(G)** Comparison of the proportion of TAM subpopulation cells in different clinical feature groups.

Myeloid cells were extracted and re-normalized for clustering analysis, resulting in the identification of 9 cell clusters at a resolution of 0.3 (**Figure 4A**). Cell types for each cluster were annotated based on myeloid subpopulation markers and highly expressed genes (**Figures 4B, C**). This analysis identified four TAM subpopulations (APOE+TAM, IL1B+TAM, CXCL10+TAM, and HSP+TAM), two monoocyte subpopulations (CD14+Mono and CD16+Mono), and two dendritic cell subpopulations (cDC and LAMP3+DC; **Figure 4D**). Characteristic genes for each cell subpopulation were identified using FindAllMarkers (**Supplementary Table S6**). The APOE+TAM subpopulation exhibited high expression of APOE, APOC1, C1QB, and C1QA, with specific expression of M2 macrophage markers MRC1, CD163, and MSR1, indicating an M2 bias. The IL1B+TAM subpopulation showed high expression of IL1B, CCL3, and CXCL12, indicating an M1 bias. The CXCL10+TAM subpopulation expressed high levels of CXCL10, ISG15, and CCL2, also indicating an M1 bias. The HSP+TAM subpopulation exhibited high expression of HSPB1, HSPA6, and HSPA1A, with no clear bias (**Figure 4E**). The distribution proportions of cell subpopulations varied among samples (**Figure 4F**; **Supplementary Table S7**). In patients aged 60 and above, the proportion of APOE+TAM cells (within the myeloid cell population) was significantly higher compared to those under 60. The proportion of IL1B+TAM cells was higher in alcohol-consuming patients than in non-drinkers, while the proportions of CXCL10+TAM and HSP+TAM cells showed no significant differences across different clinical feature groups (**Figure 4G**).

Gene set enrichment analysis (GSEA) for KEGG and GOBP pathways was performed on each myeloid cell subpopulation. The pathways related to tyrosine metabolism, galactose metabolism, and pyruvate metabolism, as well as biological processes such as receptor-mediated endocrine signaling and organic hydroxy compound metabolism, were significantly activated in the APOE+TAM cells. In contrast, biological processes related to wound healing, response to injury, and leukocyte migration were significantly activated in IL1B+TAM cells. The CXCL10+TAM cells exhibited significant activation of biological processes related to the response to viruses, negative regulation of viral genome replication, and modulation of responses to biological stimuli. Additionally, biological processes such as protein folding, response to temperature stimuli, and response to heat were significantly activated in HSP+TAM cells (**Supplementary Figure S7**; **Supplementary Table S8**).

### Characterization of NK cell interactions with TAM cells based on ligand-receptor interactions

**Figure 5:**
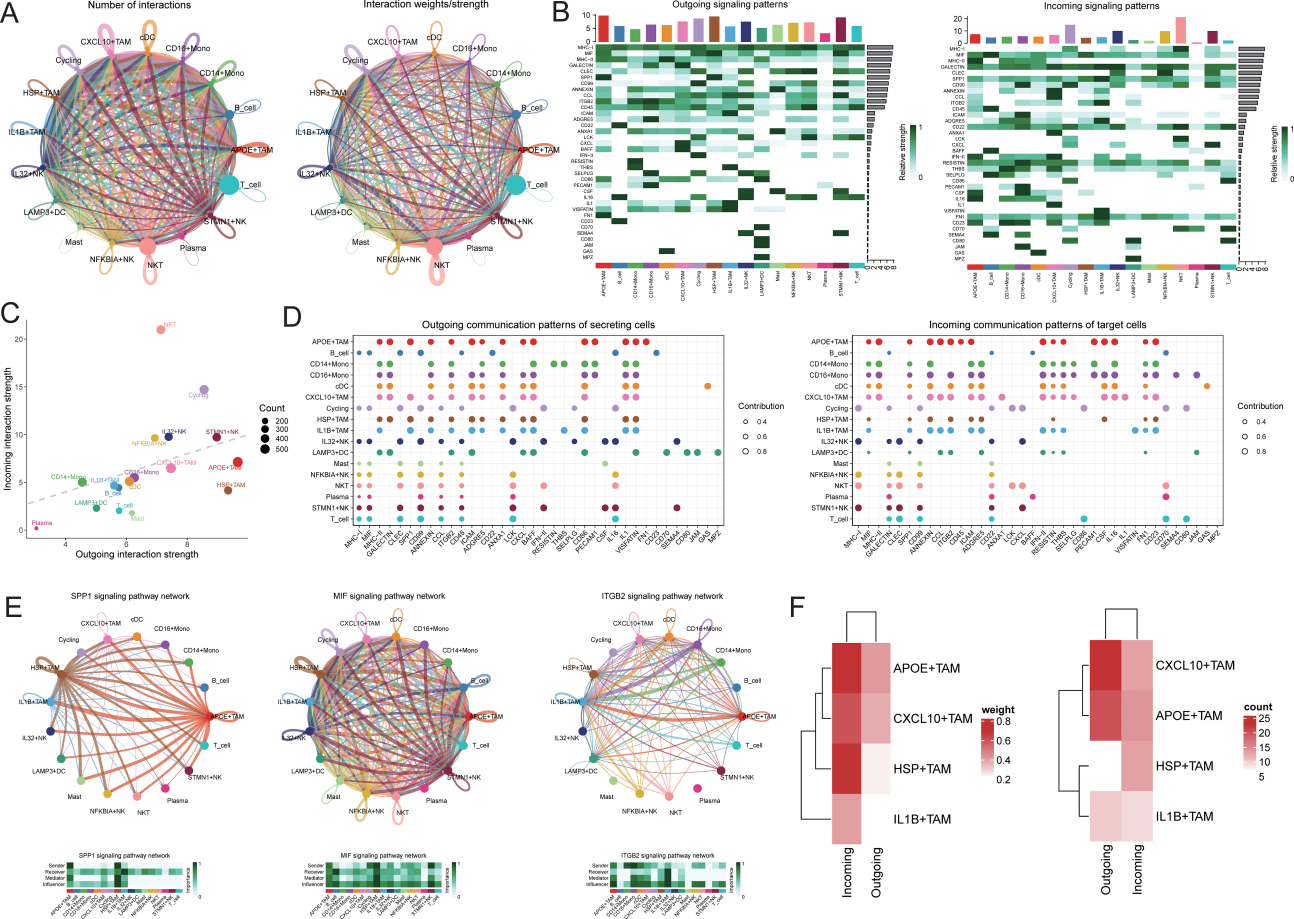
Cell communication analysis. **(A)** Receptor-ligand pairs/communication strength between different cell populations, the size of the dot represents the corresponding cell number, the more cells the bigger the dot, the thickness of the line represents the receptor-ligand pairs/communication strength between different cell populations, the more the number of ligands/communication strength the thicker the line, the color of the line is consistent with the color of the signal sender; **(B)** signaling dominant statistical heat map, heat map of the outgoing signal contribution of each pathway in the outgoing/incoming mode; **(C)** signaling dominant statistical dot map, the color of the dot represents the different cell populations, the size of the dot is proportional to the number of ligands and receptors inferred for each cell population, the x-axis and y-axis represent the strength of the cell population as a signal sender and receiver, respectively; **(D)** dot map of the outgoing signal contribution of each pathway in the outgoing/incoming mode, the horizontal axis represents each signaling pathway, the vertical axis represents each type of cell, and the contribution of each type of cell to the outgoing/incoming signal of a certain pathway; **(E)** network diagram of the signaling pathway; **(F)** heat map of the receptor-ligand pairs/communication strength between IL32+NK cells and TAM cell subsets.

To further elucidate the communication differences between cell subpopulations, communication analysis was performed using the CellChat package. A broad spectrum of cell communication was observed among the various cell groups (**Figures 5A-D**). We focused on the TAM subpopulations communicating with IL32+NK cells, finding that the interactions primarily concentrated on signaling pathways such as SPP1, MIF, and ITGB2 (**Figure 5E**). We extracted the number of ligand-receptor pairs and communication strength between IL32+NK and the four TAM subpopulations. The results revealed that both as signal senders and receivers, IL32+NK cells exhibited more extensive interactions with the APOE+TAM and CXCL10+TAM subpopulations (**Figure 5F**).

### Validation of proximity of interactive cells in three-dimensional space

**Figure 6:**
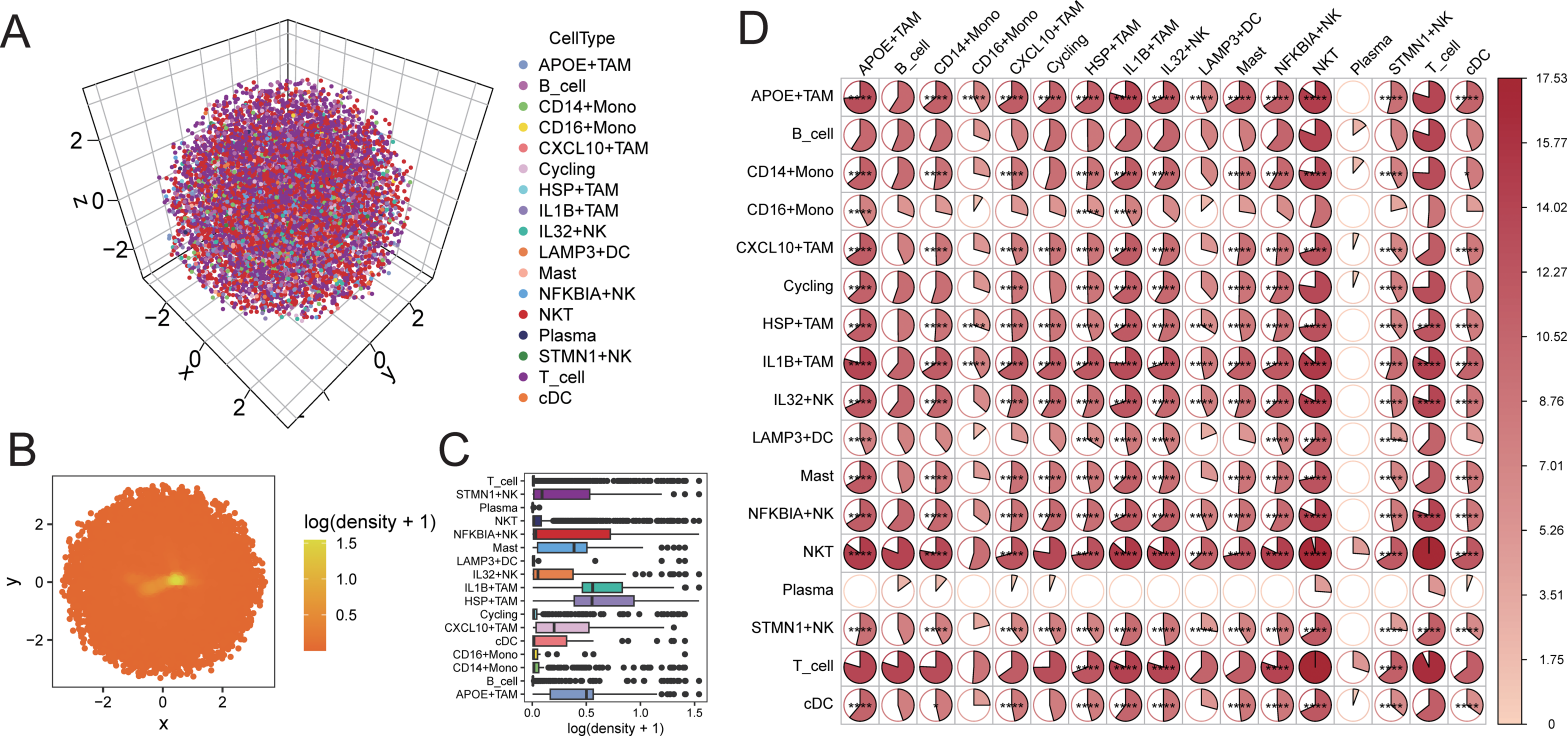
CSOmap verifies the accessibility of cells in three-dimensional space. **(A)** distribution of cells in three-dimensional space; **(B)** distribution density of cells on a two-dimensional plane; **(C)** statistical box plot of cell population distribution density; **(D)** heat map of accessibility of cell populations inthree-dimensional space (*P < 0.05, **P < 0.01, ***P < 0.001, ****P < 0.0001).

Using CSOmap, we explored the three-dimensional spatial proximity of NK cell subpopulations and TAM cell subpopulations inferred from transcriptomic data (**Figures 6A-C**). This analysis further confirmed the extensive interactions between NK cell subpopulations and TAM cell subpopulations, with particularly high proximity observed between IL32+NK and the APOE+TAM and IL1B+TAM subpopulations (**Figure 6D**).

### Immunofluorescence validation of protein expression levels

**Figure 7:**
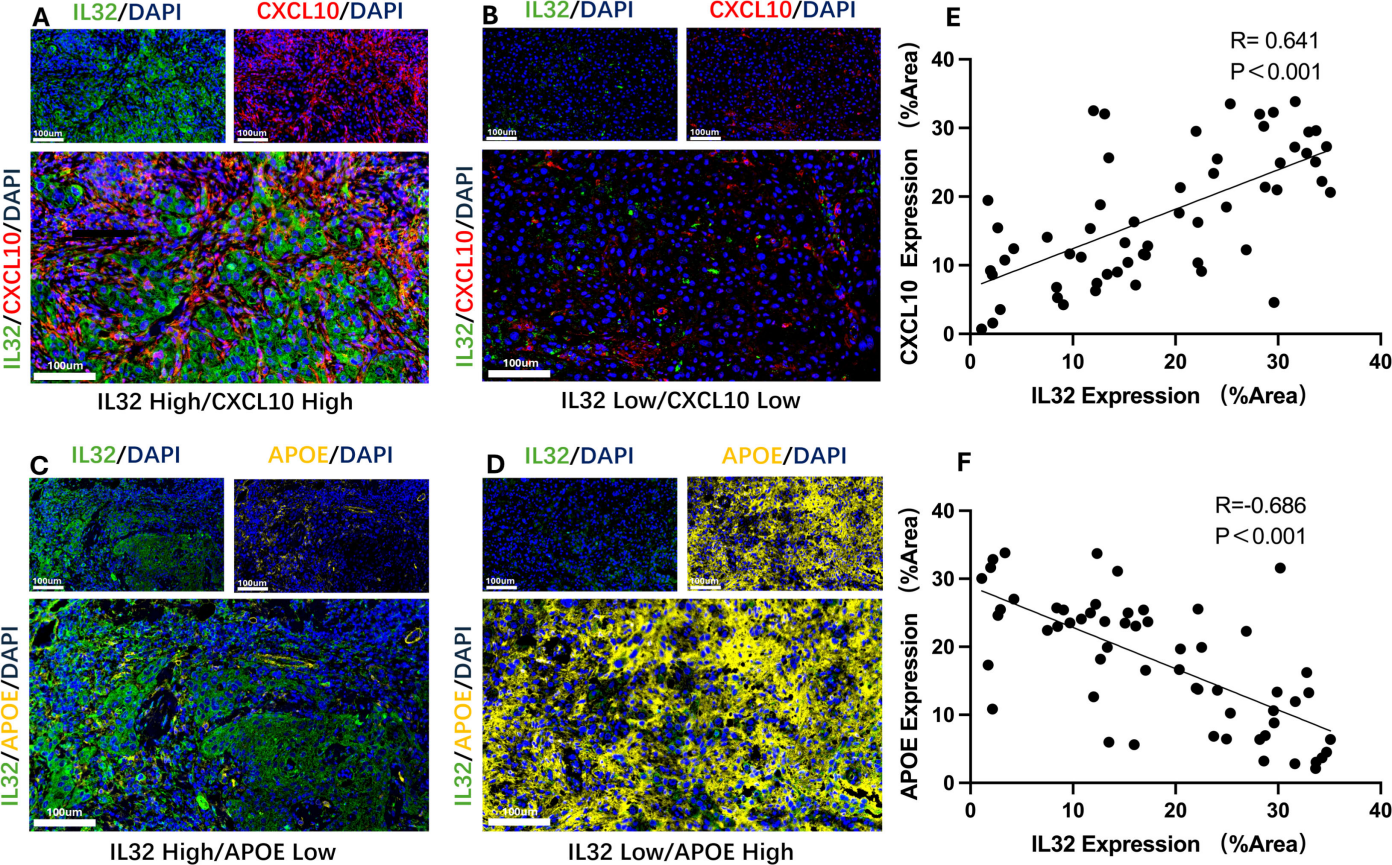
Representative immunofluorescence images and correlation analysis. **(A, B)** Representative immunofluorescence images showing high-level **(A)** and low-level **(B)** co-expression of IL32 and CXCL10; **(C)** Scatter plot demonstrating a significant positive correlation between IL32 and CXCL10 expression in HNSCC tissue samples. (*P* < 0.001, R = 0.641). **(D, E)** Representative immunofluorescence images showing high-level IL32 with low-level APOE and low-level IL32 with high-level APOE; **(F)** Scatter plot demonstrating a significant negative correlation between IL32 and APOE expression in HNSCC tissue samples. (*P* < 0.001, R = –0.686).

To further validate the interaction between IL32+NK cells and the APOE+ and CXCL10+TAM subpopulations, we performed immunofluorescence analysis to assess the expression patterns of the corresponding proteins. The results revealed a significant positive correlation between IL32 and CXCL10 expression in the majority of HNSCC tissue samples (*P* < 0.001, R = 0.641; **Figures 7A, B, E**), indicating a potential cooperative relationship. Conversely, IL32 expression was significantly negatively correlated with APOE expression (*P* < 0.001, R = –0.686; **Figures 7C, D, F**), suggesting a mutually exclusive expression pattern between IL32+NK cells and APOE+TAM.

### Construction of prognostic signature based on cell interaction mechanisms

**Figure 8:**
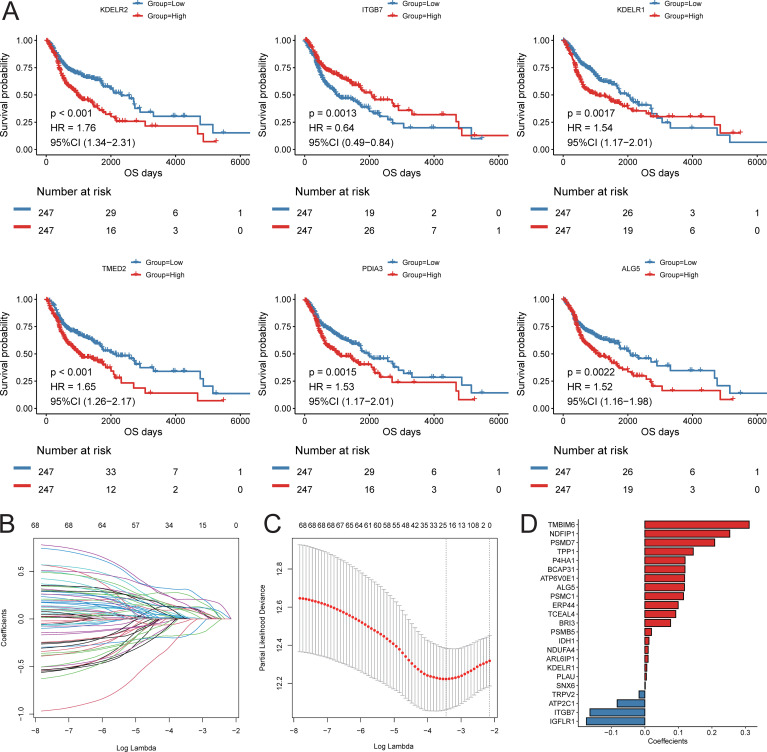
Construction of prognostic signature. **(A)** KM survival curve of top6 prognostic genes; **(B)** LASSO regression independent variable change track, the horizontal axis represents the logarithm of the independent variable Lambda, and the vertical axis represents the coefficient of the independent variable; **(C)** LASSO regression confidence interval of each Lambda; **(D)** LASSO regression coefficient of key prognostic factors.

Based on the results of cell communication and three-dimensional spatial proximity analysis, we selected 620 characteristic genes from the APOE+TAM subpopulation that had significant interactions with the IL32+NK subpopulation and were spatially accessible as candidate genes. Univariate Cox regression analysis conducted on these 620 candidate genes revealed that 69 of them were significantly associated with the prognosis of HNSC patients, including KDELR2, ITGB7, KDELR1, TMED2, PDIA3, and ALG5 (**Figure 8A**; **Supplementary Table S9**). Subsequently, LASSO-Cox regression analysis was performed on these 69 genes. Using 10-fold cross-validation under optimal conditions, we determined the penalty parameter (λ) for the model, identifying 23 key prognostic factors that influence patient survival (**Figures 8B-D**; **Supplementary Table S10**). Based on the expression levels of these 23 key prognostic factors and their corresponding weights, we constructed a signature to evaluate the prognosis of each patient, represented by the following formula:

Score = ITGB7 * (-0.164) + KDELR1 * (0.006) + ALG5 * (0.118) + ERP44 * (0.099) + BRI3 * (0.077) + TMBIM6 * (0.311) + BCAP31 * (0.120) + ATP6V0E1 * (0.119) + PLAU * (0.005) + NDUFA4 * (0.010) + IGFLR1 * (-0.175) + PSMD7 * (0.209) + PSMC1 * (0.115) + NDFIP1 * (0.253) + SNX6 * (0.001) + PSMB5 * (0.020) + TCEAL4 * (0.092) + ARL6IP1 * (0.010) + TRPV2 * (-0.017) + P4HA1 * (0.120) + TPP1 * (0.144) + ATP2C1 * (-0.083) + IDH1 * (0.012).

**Figure 9:**
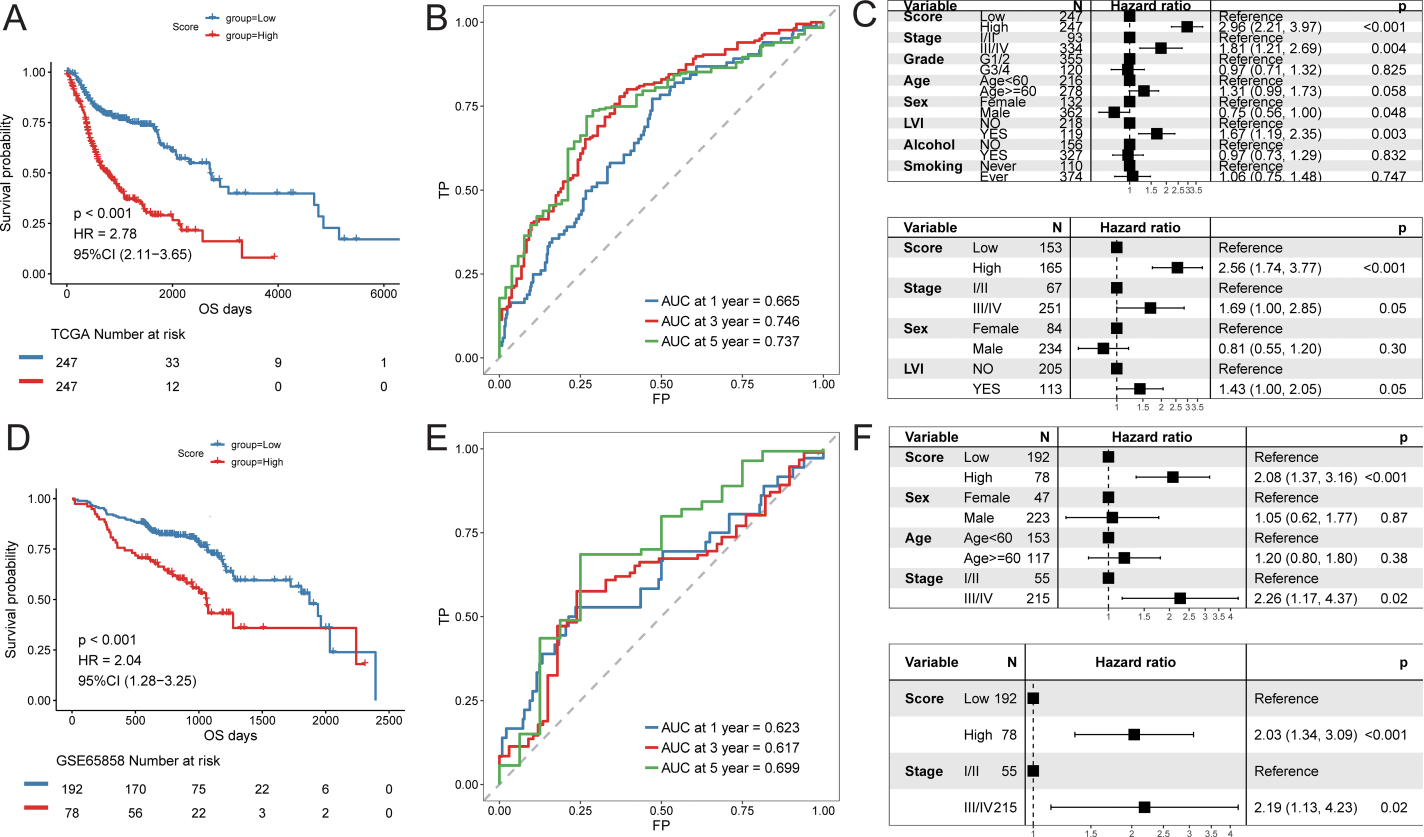
Predictive efficacy of prognostic signature. Training cohort (TCGA-HNSC): **(A)** Kaplan–Meier survival analysis comparing high- and low-risk groups; **(B)** time-dependent ROC curves at 1, 3, and 5 years; **(C)** univariate and multivariate Cox regression analyses of the risk score and clinical variables; Validation cohort (GSE65858): **(D)** Kaplan–Meier survival analysis; **(E)** time-dependent ROC curves; **(F)** univariate and multivariate Cox regression analyses.

Using the constructed prognostic signature, we calculated the risk scores for each patient in the training set and divided them into high-risk and low-risk groups. Kaplan-Meier curve analysis and log-rank tests indicated that patients in the high-risk group had a significantly shorter OS (*p* < 0.05, **Figure 9A**). The predictive AUC values for the samples at 1 year, 3 years, and 5 years were 0.665, 0.746, and 0.737, respectively (**Figure 9B**), suggesting that the score can effectively characterize the OS of the samples. We then explored the independence of the prognostic signature within the training set. Cox regression models, both univariate and multivariate, were constructed based on the prognostic signature and clinical characteristics. The results indicated that the prognostic signature is an independent prognostic factor (*p* < 0.05, **Figure 9C**).To assess the reliability of the prognostic signature, we used GSE65858 as an independent validation cohort. Patients were divided into high-risk and low-risk groups based on the prognostic signature risk scores. The overall survival rate of the high-risk group was also significantly lower than that of the low-risk group (**Figure 9D**). The predictive AUC values for the GSE65858 samples at 1 year, 3 years, and 5 years were 0.623, 0.617, and 0.699, respectively (**Figure 9E**). Consistent results were obtained from the Cox regression models based on the prognostic signature and clinical characteristics, further supporting the notion that the prognostic signature is an independent prognostic factor (**Figure 9F**).

### Potential applications of the cell interaction prognostic signature

**Figure 10:**
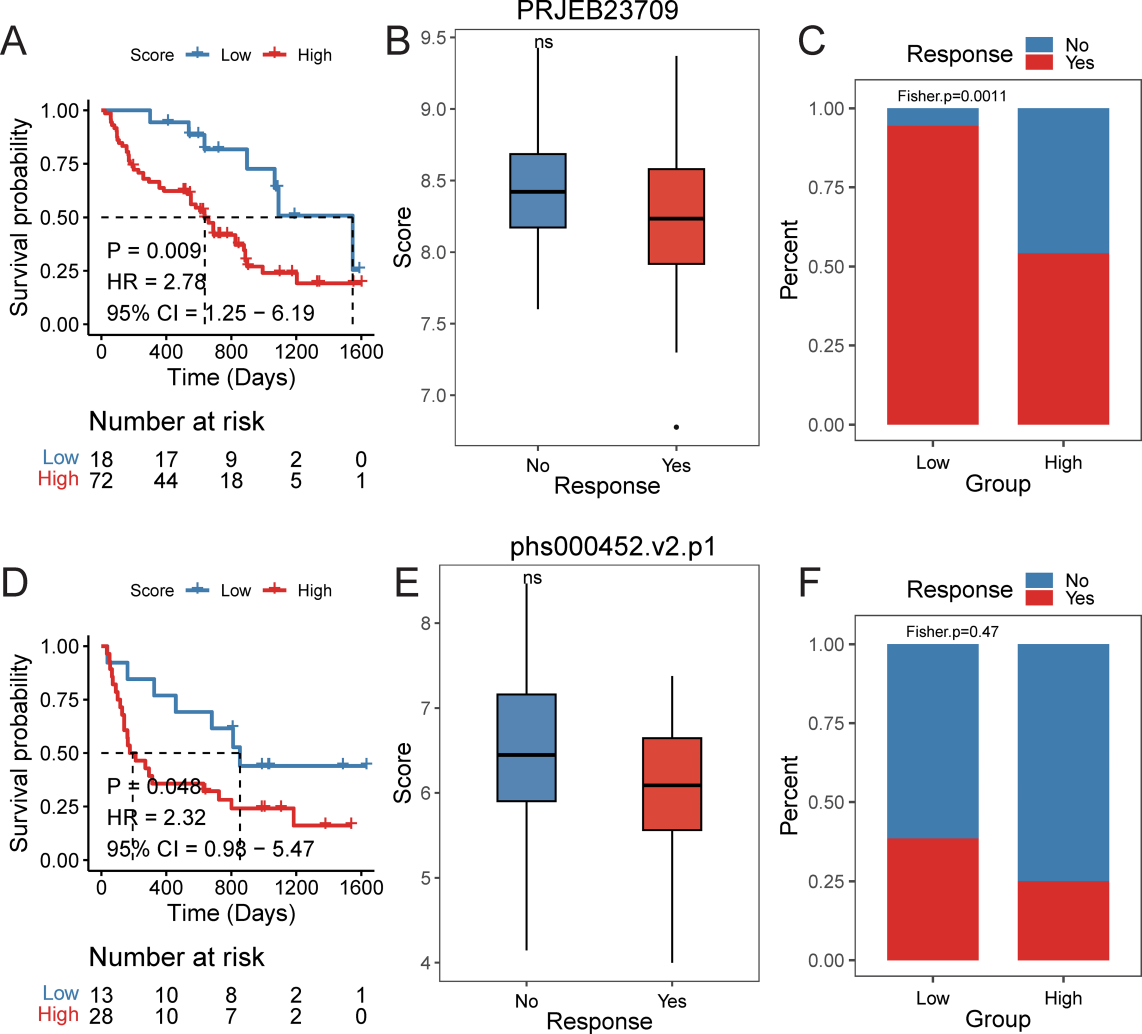
The efficacy of prognostic signature in immune data set. PRJEB23709 cohort: **(A)** Kaplan–Meier survival curves comparing overall survival between high- and low-risk groups, with statistical significance assessed by the log-rank test (P < 0.05). **(B)** Distribution of risk scores across different immunotherapy response groups. **(C)** Distribution of immunotherapy response status between high- and low-risk groups. phs000452.v2.p1 cohort: **(D)** Kaplan–Meier survival curves comparing overall survival between high- and low-risk groups, with statistical significance assessed by the log-rank test (P < 0.05). **(E)** Distribution of risk scores across different immunotherapy response groups. **(F)** Distribution of immunotherapy response status between high- and low-risk groups.

Furthermore, we explored the predictive efficacy of the prognostic signature for sample prognosis in the immunotherapy cohorts PRJEB23709 and phs000452.v2.p1. Similarly, patients with a high score had a significantly lower overall survival rate compared to those with a low score (**Figures 10A-D**). Although not statistically significant, patients in the responder group to immunotherapy had lower scores than those in the non-responder group (**Figure 10E**). The proportion of patients responding to immunotherapy was higher in the low score group compared to the high score group (**Figure 10F**).

## Discussion

HNSCC is one of the most common malignant tumors, characterized by extremely high malignancy, poor prognosis, and a low 5-year survival rate (22). The emergence of cancer immunotherapy has transformed treatment models and is receiving increasing attention (5, 23). In the HNSCC patients analyzed, elevated expression of CD16 and CD64 in tumor tissue was associated with improved prognosis, whereas high CD163 expression correlated with reduced five-year survival. CD16, CD64, and CD163 are markers of NK cells and TAMs in the tumor microenvironment, respectively. This study found that CD16 and CD64 were highly expressed in early-stage disease and well-differentiated tumors, whereas CD163 was upregulated in late-stage and poorly differentiated tumors. These findings suggest that NK cells and TAMs in the tumor immune microenvironment may be closely associated with patient prognosis.

NK cells are a key component of the innate immune system and serve as the first line of defense against cancer cell invasion (24). M1 macrophages, another crucial part of the innate immune system, exhibit versatile functions. They can phagocytize and kill tumor cells, contributing to immune defense, immune homeostasis, immune surveillance, and antigen presentation. However, the tumor microenvironment is primarily dominated by M2 macrophages, which suppress immune responses and promote tumor progression (25). Although there have been many studies on NK cells or TAMs, research on their comprehensive effects and potential clinical applications remains limited.

The resulting 23-gene prognostic signature, termed the NK and TAM Composite Index (CINT), effectively stratified patients into high- and low-risk groups across multiple cohorts. The area under the curve (AUC) values for overall survival prediction (0.62–0.74 across time points and datasets) indicate moderate predictive accuracy, comparable to other recently reported immune-related prognostic models in HNSCC (26). While this performance is encouraging for a biologically driven signature grounded in cellular crosstalk, its clinical utility would benefit from further refinement and validation. Although the immunotherapy cohorts used for validation were relatively small, the consistent performance of CINT across independent datasets (PRIEB23709 and phs000452.v2.p1) highlights its potential generalizability and supports the need for evaluation in larger, multi-center immunotherapy-specific cohorts. Moreover, future studies incorporating calibration and decision-curve analyses will be essential to rigorously assess its net benefit relative to established clinical parameters and prognostic tools. Importantly, the CINT signature is uniquely grounded in the specific ligand–receptor crosstalk between NK cells and TAM subsets, moving beyond purely correlative associations to provide a more mechanistic explanation of tumor microenvironment dysfunction. This biological foundation not only strengthens its prognostic relevance but also offers deeper insights into actionable immune escape pathways and potential therapeutic targets.

Our study identifies the IL32+NK–APOE+TAM axis as a potential therapeutic target in head and HNSCC. Reprogramming immunosuppressive APOE+TAM toward an antitumor phenotype—for example, with CSF1R inhibitors or CD40 agonists (27, 28)—may help restore immune surveillance. Conversely, boosting the activity and recruitment of beneficial IL32+NK cells through IL-15 (29) superagonists or approaches that enhance IL-32 signaling could shift the balance toward antitumor immunity. Furthermore, emerging strategies such as chimeric antigen receptor-engineered NK (CAR-NK) cells represent a promising avenue to enhance NK cell cytotoxicity and persistence within the tumor microenvironment, potentially acting synergistically with TAM-targeted therapies to overcome immunosuppression (30, 31).

Numerous studies have highlighted the diverse roles of IL-32 in various cancers. In some tumors, IL-32 contributes to cancer progression by modulating key signaling pathways, including NF-κB, STAT3, and MAPK (32, 33). In contrast, in other cancers, IL-32 can promote tumor cell apoptosis and enhance the toxicity of NK cells, thereby exerting an inhibitory effect on tumor growth (32–34). One study reported that elevated expression of IL-32 is correlated with a worse prognosis in patients with HNSCC (35). In our research, we found that the characteristic score of the IL32+NK cell subpopulation is associated with prognosis, where patients in the high-feature group experienced better outcomes. The gene expression profile of this subpopulation revealed that high expression is linked to genes involved in responses to viruses and external stimuli, as well as the regulation of T cell activation. This suggests that IL32+NK cells may play a crucial role in immune surveillance and regulation within the immune microenvironment of head and neck squamous cell carcinoma. Their enhanced ability to respond to viral and external challenges likely enables them to more effectively recognize tumor cells, while their role in T cell activation may bolster the overall immune response, thereby inhibiting tumor progression and leading to improved prognosis.

Conversely, the TAM subpopulations exhibited functional heterogeneity, including subtypes with differing tendencies, such as APOE+TAM (M2-like) and IL1B+TAM (M1-like). Notably, certain TAM subpopulations showed variation in proportion among patients with different clinical characteristics. For instance, the proportion of APOE+TAM cells was significantly higher in patients aged 60 and above compared to those under 60, and the IL1B+TAM cell proportion was greater in alcohol-consuming patients than in non-drinkers. These findings suggest that clinical characteristics may influence the distribution of TAM subpopulations, which, in turn, can impact tumor progression and patient prognosis. For instance, in elderly patients, chronic inflammatory states accumulated over time may lead TAMs to shift predominantly toward the M2 subtype (36, 37), potentially increasing the risk of tumor progression. Notably, several studies have shown that prolonged alcohol exposure activates monocytes and macrophages, leading to an increased production of pro-inflammatory cytokines, including TNF-α, IL-1, IL-6, and the chemokine IL-8 (38). This may explain the higher ratio of IL1B+TAM observed in alcohol consumers. The complex mechanisms underlying this effect could indirectly influence tumor development and prognosis, warranting further investigation to confirm these hypotheses.

The spatial organization of cells is closely linked to their functions and behaviors, including cell-to-cell interactions. In our study, we characterized the interactions between NK cells and TAM cells based on ligand-receptor interactions. Using the CSOmap algorithm, we inferred the three-dimensional accessibility and communication information between NK cell subpopulations and TAM cell subpopulations, demonstrating extensive interactions. The results revealed that IL32+NK cells interacted more broadly with APOE+TAM and CXCL10+TAM subpopulations, primarily focusing on signaling pathways involving SPP1, MIF, and ITGB2. Our immunofluorescence results further confirmed that IL32 expression in HNSCC was positively correlated with CXCL10 expression and negatively correlated with APOE expression. SPP1, also known as osteopontin (OPN), is highly expressed in various malignant tumors (39). In head and neck squamous cell carcinoma, it binds to specific receptors, activating the PI3K/Akt and MAPK signaling pathways. This activation promotes the transformation of normal cells into malignant ones, enhances the aggressive behavior of tumor-related cells, and ultimately contributes to tissue infiltration and distant metastasis (40, 41). Tumor cells also express high levels of MIF, which enables malignant tumors to evade immune surveillance by inhibiting NK cell-mediated detection and clearance (8, 42). Furthermore, MIF promotes tumorigenesis by preventing ferroptosis in macrophages and driving them toward an M2-like phenotype, further supporting tumor progression (43, 44). In tumors, β2 integrin plays a key role in cell adhesion, stromal remodeling, and signal transduction, facilitating interactions among tumor cells and between tumor cells and the tumor microenvironment (45). These activities promote infiltration, angiogenesis, and tumor-specific immune responses. Likewise, ITGB2 is closely associated with tumor progression, contributing to cancer development, metastasis, and invasion (46). For example, one study demonstrated that ITGB2 promotes OSCC proliferation by enhancing glycolytic activity in cancer-associated fibroblasts (CAFs) through the PI3K/AKT/mTOR pathway (47). In summary, prior experimental studies have already demonstrated that TAMs can directly suppress or reprogram NK cells via contact-dependent mechanisms and soluble mediators, and that molecules such as MIF and SPP1 are implicated in shaping tumor-promoting myeloid phenotypes and inhibiting anti-tumor lymphocyte functions (15, 48, 49).

Despite the comprehensive nature of our analysis, several limitations should be noted. The experimental validation cohort was relatively small (n = 58) and derived from a single institution, which may introduce selection bias, although this was partly mitigated by validation in large public datasets. Future multicenter prospective studies with larger cohorts are needed to reduce bias and validate the robustness of our conclusions. The 23-gene CINT signature, while demonstrating robust prognostic performance, presents practical challenges for clinical translation due to the complexity and cost of multi-gene detection. Future optimization should therefore focus on developing streamlined formats, such as a reduced core-gene panel identified by machine learning, or simplified detection platforms (e.g., NanoString, targeted RT-qPCR, or minimal gene/protein classifiers), to maintain comparable prognostic value while improving feasibility in routine diagnostics. In addition, the immunotherapy validation cohorts (n = 90 and n = 41) were modest in size, potentially limiting the generalizability of our findings across diverse patient populations and treatment settings, highlighting the need for validation in larger multi-center cohorts. Finally, our computational analyses suggested that NK cells interact with TAMs and exhibit spatial correlation; however, the underlying mechanisms still require functional validation. Future studies, including co-culture and blocking experiments, will be conducted to further confirm these NK–TAM interactions. Moreover, although this study primarily focused on NK–TAM interactions, we do not exclude the potential contributions of other immune cell populations within the tumor microenvironment.

## Data Availability

The datasets presented in this study can be found in online repositories. The names of the repository/repositories and accession number(s) can be found in the article/**Supplementary Material**.

## Supplementary material

Supplementary Figure 1 GSEA enrichment analysis of myeloid cell subsets. A KEGG enrichment analysis NES heat map of each subpopulation; B GOBP enrichment analysis NES heat map of top5 subpopulations; C GOBP enrichment analysis rank map of top5 subpopulations.
