# Supplementary figures and images for: Subtype-specific NK cell-TAM interactions drive a novel prognostic signature in HNSCC
Source: Front Immunol. 2025 Oct 17;16:1676878. doi: 10.3389/fimmu.2025.1676878 (PMC12575298; doi:10.3389/fimmu.2025.1676878)

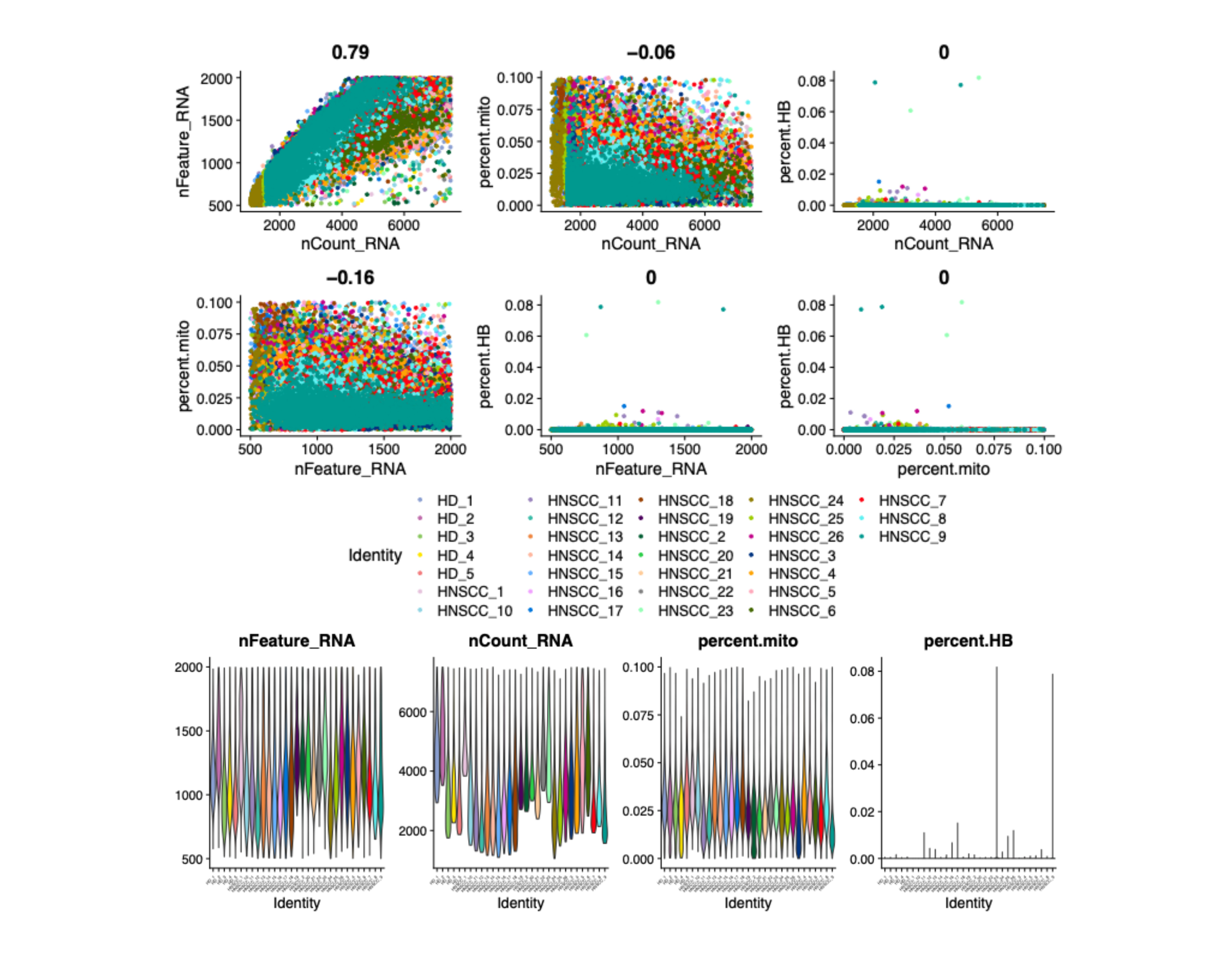

Supplement: Supplementary Figure 1 — GSEA enrichment analysis of myeloid cell subsets. A KEGG enrichment analysis NES heat map of each subpopulation; B GOBP enrichment analysis NES heat map of top5 subpopulations; C GOBP enrichment analysis rank map of top5 subpopulations. [file Image1.tif]

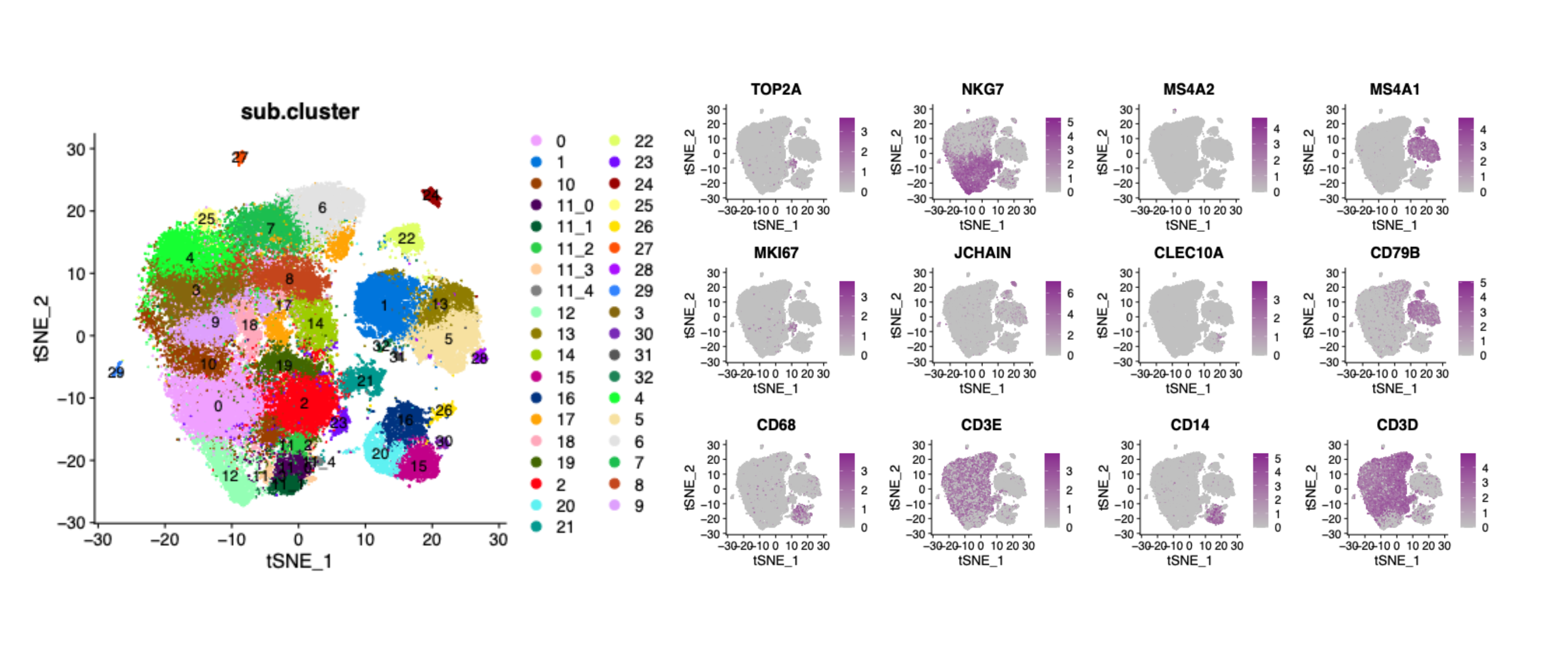

Supplement: Supplementary file 2 [file Image2.tif]

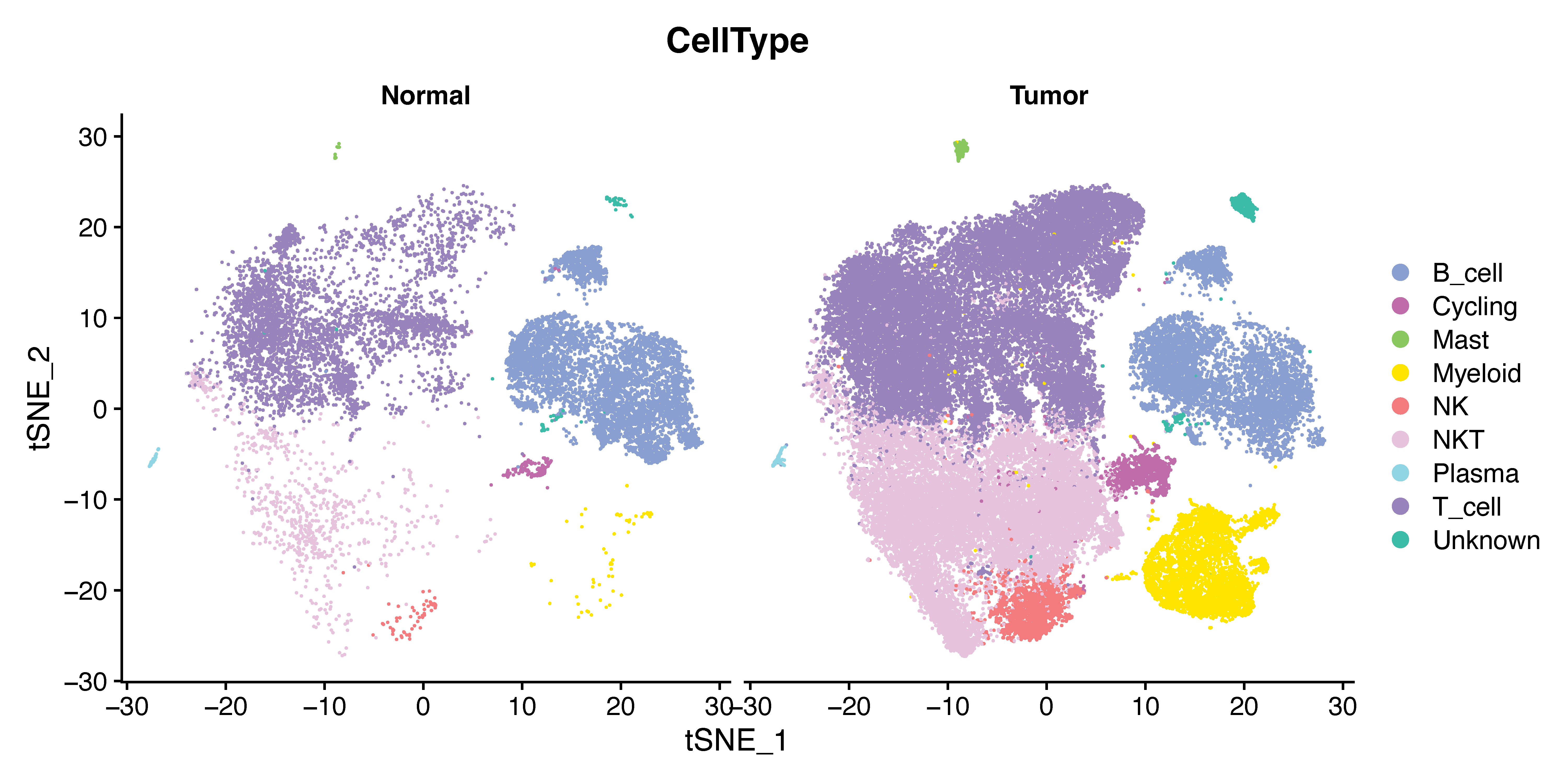

Supplement: Supplementary file 3 [file Image3.tiff]

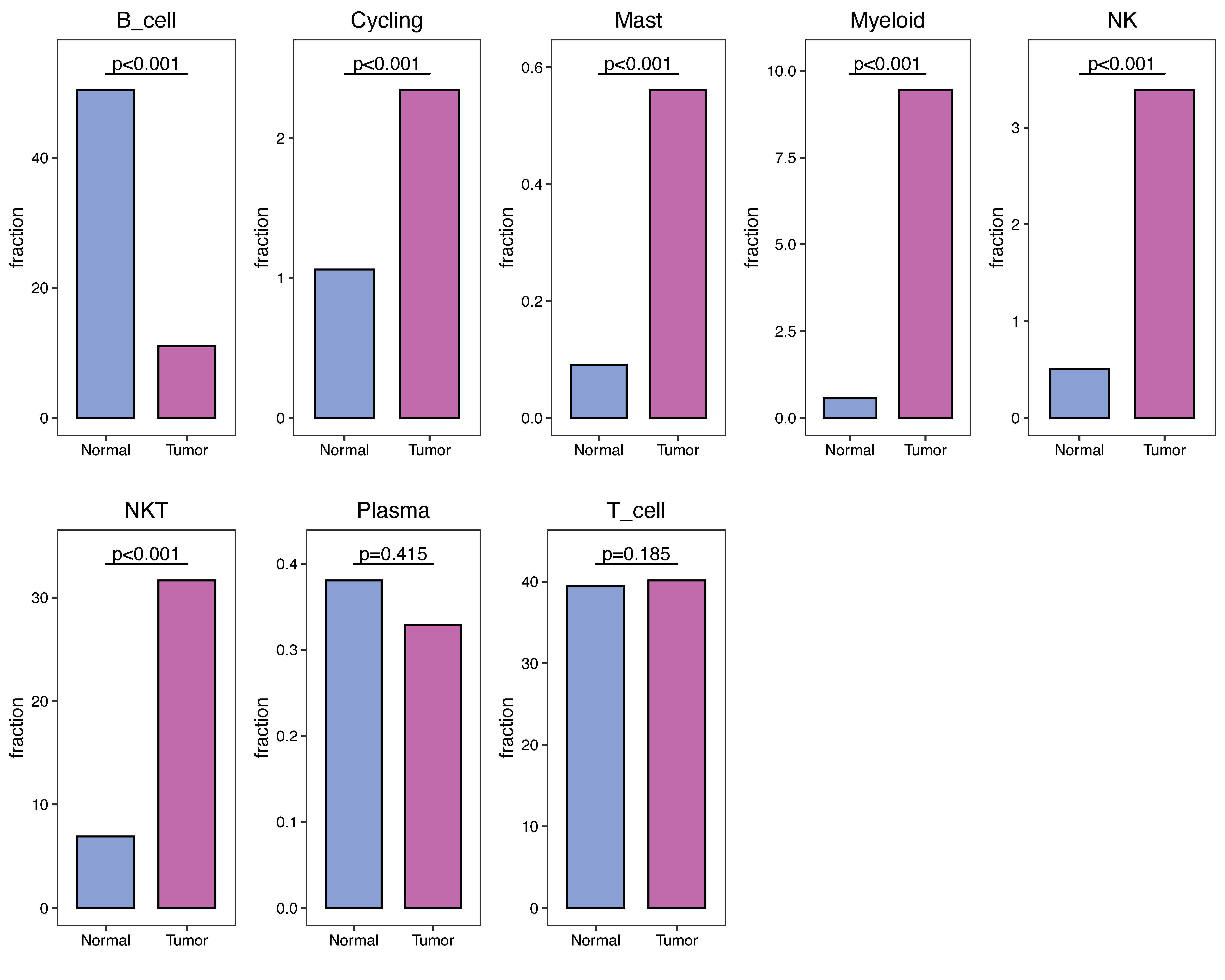

Supplement: Supplementary file 4 [file Image4.tiff]

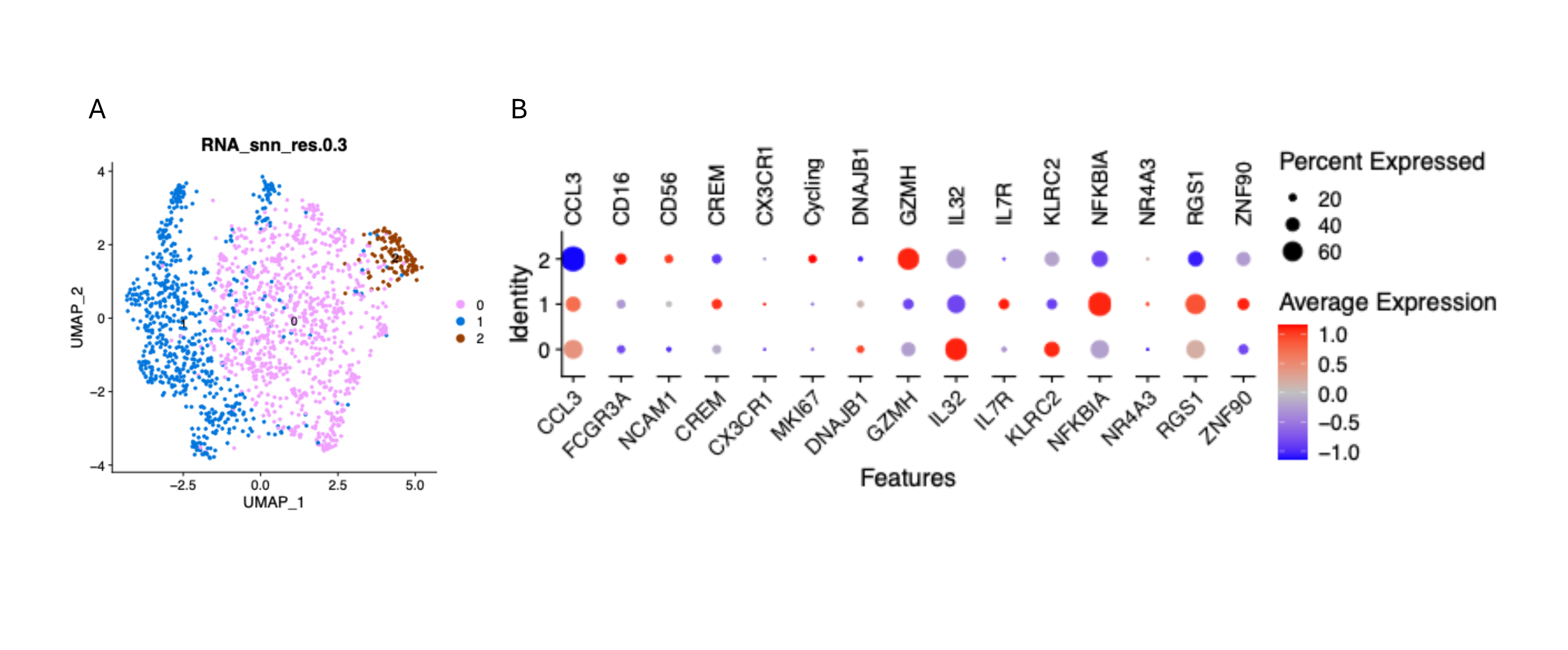

Supplement: Supplementary file 5 [file Image5.tif]

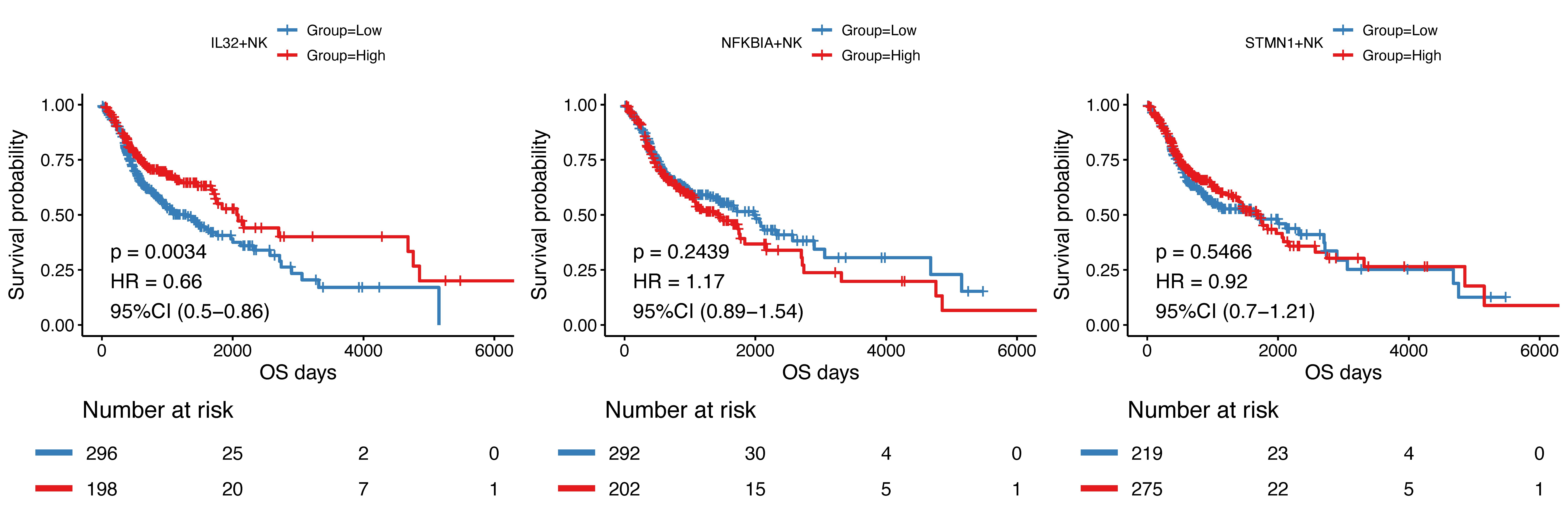

Supplement: Supplementary file 6 [file Image6.tiff]
